# Public Belief in the Maternal Health Benefits of Breastfeeding — United States, 2018 and 2021

**DOI:** 10.5888/pcd20.230010

**Published:** 2023-08-24

**Authors:** Ellen O. Boundy, Jennifer M. Nelson, Ruowei Li

**Affiliations:** 1 Division of Nutrition, Physical Activity, and Obesity, National Center for Chronic Disease Prevention and Health Promotion, Centers for Disease Control and Prevention, Atlanta, Georgia; 2 US Public Health Service, Rockville, Maryland

## Abstract

The objective of this study was to better understand US public awareness of maternal health benefits of breastfeeding. Data from the 2018 and 2021 SummerStyles surveys were analyzed to explore public belief in select maternal benefits of breastfeeding. As in 2018, in 2021 a low percentage of respondents believed that breastfeeding protects the mother against breast cancer (23.9%), high blood pressure (15.5%), or type 2 diabetes (15.4%), with male, older, and unmarried respondents less likely to believe in these protective effects. More public awareness of maternal benefits of breastfeeding might help increase demand for breastfeeding-supportive programs and policies.

**Summary**

**What is already known on this topic?**

Most members of the US public are aware of the health benefits of breastfeeding for infants.

**What is added by this report?**

Data from the 2018 and 2021 SummerStyles public opinion survey show that most US adults are unaware of the protective effects of breastfeeding against breast cancer, high blood pressure, and type 2 diabetes. Awareness was particularly low among respondents who were male, older, and not married.

**What are the implications for public health practice?**

Maternal health benefits of breastfeeding are well established and can be incorporated into public awareness campaigns. Increased awareness of maternal and infant benefits of breastfeeding could lead to more support for improving programs and policies for breastfeeding families.

## Objective

Breast milk is the recommended nutrition for most infants. Its benefits for infant health, growth, and development are well established, including decreased risk of developing asthma, type 1 diabetes, and obesity (1). Health benefits for mothers who breastfeed are also significant. A 2018 systematic review identified breastfeeding as associated with decreased risk for maternal development of high blood pressure, type 2 diabetes, breast cancer, and ovarian cancer (2).

Although public awareness of some of the benefits of breastfeeding for infants has been documented (3), evidence on public knowledge and perceptions around the effect of breastfeeding on maternal health later in life is limited (4). We aimed to better understand beliefs among the general public in the US around the maternal health benefits of breastfeeding by examining data available for 3 outcomes: breast cancer, high blood pressure, and type 2 diabetes.

## Methods

We conducted a cross-sectional study of data from Porter Novelli SummerStyles, an online marketing survey collecting health-related opinions (5). The survey panel is representative of the noninstitutionalized US adult population, with members randomly recruited by mail using probability-based sampling by address, and continuously replenished to maintain approximately 60,000 panelists. Data did not include identifiable information and were determined exempt by the Centers for Disease Control and Prevention’s Institutional Review Board.

We examined questions from 2018 and 2021 on participants’ perceptions of the protective effects of breastfeeding against breast cancer, high blood pressure, and type 2 diabetes (2021 only). Each stated, “If a mother breastfeeds her baby, she may be less likely to develop [condition] later in her life.” Responses were collapsed into 3 categories: agree (somewhat agree/strongly agree), neutral (neither agree nor disagree), and disagree (somewhat disagree/strongly disagree). Surveys missing responses (27 in 2018; 35 in 2021) were excluded.

We calculated weighted percentages for each outcome and performed χ^2^ tests to assess differences in responses from 2018 to 2021. Stratified analyses were conducted for 2021 by characteristics associated with breastfeeding, including gender, age in years (18–29, 30–44, 45–59, ≥60), race and ethnicity (Black or African American, non-Hispanic; Hispanic; other race, non-Hispanic; White, non-Hispanic; ≥2 races, non-Hispanic), education (high school graduate or less, some college, bachelor’s degree or higher), annual household income (<$25,000, $25,000–$49,999, $50,000–$99,999, ≥$100,000), employment (working, not working), marital status (married, not married), metro status (metro, nonmetro), and region (Northeast, Midwest, South, West).

Adjusted odds ratios and 95% CIs were calculated by using multivariable logistic regression, with each respondent characteristic as exposures and each maternal benefit as outcomes (responses dichotomized as agree or neutral/disagree), adjusted for all other characteristics.

Analyses were conducted in SAS version 9.4 using SummerStyles adult survey weights (5), which were weighted to the 2017 and 2019 US Census distributions of gender, age, annual household income, race and ethnicity, household size, education, census region, and metro status.

## Results

Response rates among panelists invited to participate in the SummerStyles survey were 73% in 2018 and 71% in 2021. Among 4,050 respondents in 2021, 23.9% believed that mothers who breastfeed are less likely to develop breast cancer, 66.7% were neutral, and 9.4% disagreed (Table 1). Sixteen percent of respondents believed that mothers who breastfeed are less likely to develop high blood pressure, 76.0% were neutral, and 8.4% disagreed. Responses for the type 2 diabetes outcome were similar at 15.4%, 74.3%, and 10.2%, respectively. Thirty percent of respondents in 2021 believed in at least 1 of the health benefits examined, and 9.3% believed all 3. From 2018 to 2021, the percentage of respondents believing in the protective effects against breast cancer and high blood pressure remained about the same (*P* = .12 and *P* = .37, respectively).

**Table 1:** Public Awareness of Maternal Health Benefits of Breastfeeding — SummerStyles, United States, 2018 and 2021^a^

Question/Year	N	% Agree (95% CI)	% Neutral (95% CI)	% Disagree (95% CI)
If a mother breastfeeds her baby, she may be less likely to develop **breast cancer** later in her life.^b^				
2018	4,061	25.4 (23.8–26.9)	62.0 (60.3–63.7)	12.6 (11.4–13.8)
2021	4,050	23.9 (22.4–25.4)	66.7 (65.0–68.5)	9.4 (8.3–10.5)
If a mother breastfeeds her baby, she may be less likely to develop **high blood pressure** later in her life.^c^				
2018	4,061	14.8 (13.5–16.1)	72.6 (71.0–74.2)	12.6 (11.4–13.8)
2021	4,050	15.5 (14.2–16.9)	76.0 (74.5–77.6)	8.4 (7.4–9.5)
If a mother breastfeeds her baby, she may be less likely to develop **type 2 diabetes** later in her life.^d^				
2021	4,050	15.4 (14.1–16.8)	74.3 (72.7–76.0)	10.2 (9.0–11.4)

a Percentages and 95% CIs are estimated using SummerStyles adult survey weights.

b Chi-square *P* = .12 comparing responses of agree to breastfeeding question in 2018 compared with 2021.

c Chi-square *P* = .37 comparing responses of agree to high blood pressure question in 2018 compared with 2021.

d Question not asked in SummerStyles 2018 survey.

In 2021, public belief in the protective effects of breastfeeding differed by respondent gender, age, race and ethnicity, education, household income, and marital status in unadjusted analyses for at least 1 of the maternal health outcomes examined (Table 2).

**Table 2:** Public Awareness of Maternal Health Benefits of Breastfeeding, by Respondent Characteristics — SummerStyles, United States, 2021^a^

Characteristic	N	Breast cancer			High blood pressure			Type 2 diabetes		
		% Agree	% Neutral	% Disagree	% Agree	% Neutral	% Disagree	% Agree	% Neutral	% Disagree
**Total**	4,050	23.9	66.7	9.4	15.5	76.0	8.4	15.4	74.3	10.2
**Gender**		*P* < .001			*P* = .001			*P* < .001		
Male	2,077	19.5	72.3	8.2	14.7	78.8	6.5	13.4	77.8	8.8
Female	1,973	28.0	61.5	10.5	16.3	73.5	10.2	17.3	71.1	11.6
**Age, y**		*P* = .47			*P* < .001			*P* < .001		
18–29	377	22.8	67.1	10.0	18.2	74.1	7.7	17.9	70.9	11.3
30–44	987	25.9	66.4	7.7	21.1	72.1	6.8	18.8	72.4	8.8
45–59	1,164	24.9	65.4	9.7	13.8	76.8	9.4	16.4	73.3	10.2
≥60	1,522	22.1	67.9	10.0	10.4	80.1	9.5	10.1	79.2	10.7
**Race and ethnicity**		*P* = .001			*P* = .001			*P* = .001		
Black or African American, non-Hispanic	306	18.2	66.2	15.6	15.9	69.0	15.1	15.3	67.2	17.5
Hispanic	408	23.7	65.0	11.2	15.1	74.8	10.1	16.0	71.6	12.5
White, non-Hispanic	3,007	24.5	67.6	7.9	15.1	77.8	7.1	14.9	76.6	8.5
≥2 races, non-Hispanic	128	36.7	52.6	10.7	21.2	70.0	8.8	22.5	67.9	9.6
Other race, non-Hispanic	201	24.8	67.5	7.7	18.1	76.9	5.0	17.5	73.9	8.6
**Education**		*P* < .001			*P* = .003			*P* < .001		
High school graduate or less	1,212	19.1	69.7	11.2	13.2	76.7	10.0	12.3	75.2	12.5
Some college	1,235	25.2	66.1	8.7	15.8	75.5	8.8	17.5	73.2	9.3
Bachelor’s degree or higher	1,603	28.5	63.8	7.8	18.0	75.8	6.2	17.3	74.4	8.3
**Annual household income, $**		*P* < .001			*P* = .57			*P* = .10		
<25,000	323	15.5	71.4	13.1	12.3	77.7	10.0	11.1	76.0	12.9
25,000–49,999	609	20.2	69.0	10.8	14.7	75.9	9.3	14.5	73.3	12.2
50,000–99,999	1,291	25.8	65.7	8.5	16.5	75.5	8.1	15.9	75.0	9.1
≥100,000	1,827	26.7	65.1	8.2	16.1	76.1	7.8	16.9	73.7	9.4
**Employment**		*P* = .16			*P* = .17			*P* = .11		
Working	2,463	24.6	66.9	8.6	16.6	75.2	8.2	16.6	73.7	9.7
Not working	1,587	22.9	66.5	10.6	13.9	77.3	8.8	13.7	75.3	10.9
**Marital status**		*P* < .001			*P* = .22			*P* = .01		
Married	2,714	27.8	63.1	9.1	16.6	75.0	8.4	17.3	73.4	9.3
Not married	1,336	18.7	71.5	9.8	14.1	77.4	8.5	13.0	75.6	11.4
**Metro status**		*P* = .34			*P* = .96			*P* = .90		
Metro area	550	25.9	66.5	7.6	15.1	76.7	8.2	14.7	75.3	10.0
Nonmetro area	3,500	23.6	66.8	9.6	15.6	76.0	8.5	15.5	74.2	10.3
**Region**		*P* = .15			*P* = .89			*P* = .07		
Northeast	753	20.3	69.8	9.9	14.6	76.6	8.8	15.4	76.2	8.4
Midwest	890	26.5	63.6	9.9	16.0	74.7	9.2	16.2	74.6	9.2
South	1,457	22.8	67.5	9.7	15.3	76.1	8.6	14.8	72.5	12.7
West	950	25.9	66.1	8.0	16.0	76.7	7.3	15.8	75.7	8.5

a Percentages are estimated using SummerStyles adult survey weights. *P* values based on χ^2^ test of weighted proportions.

After adjusting for other characteristics, respondents who were not married were less likely to believe in the benefits of breastfeeding across all 3 maternal health outcomes, compared with married respondents (Table 3). Men and those aged 60 years or older were less likely to believe in the benefits of breastfeeding against breast cancer and type 2 diabetes, compared with women and those aged 18 to 29 years. Being a high school graduate or less and living in the Northeast region were also independently associated with less belief in the benefit of breastfeeding against breast cancer, compared with respondents with a bachelor’s degree or higher and those living in the Midwest, respectively. Those who identified as multiracial non-Hispanic were more likely to believe in the protective effect for breast cancer than non-Hispanic White respondents.

**Table 3:** Adjusted Odds Ratios for Agreement With Statements on Maternal Health Benefits of Breastfeeding, by Respondent Characteristics — SummerStyles, United States, 2021^a^

Characteristic	Breast cancer		High blood pressure		Type 2 diabetes	
	% Agree	Adjusted OR (95% CI)	% Agree	Adjusted OR (95% CI)	% Agree	Adjusted OR (95% CI)
**Total**	23.9	NA	15.5	NA	15.4	NA
**Gender**						
Male	19.5	0.59 (0.50–0.71)	14.7	0.86 (0.70–1.07)	13.4	0.71 (0.58–0.88)
Female	28.0	1 [Reference]	16.3	1 [Reference]	17.3	1 [Reference]
**Age, y**						
18–29	22.8	1 [Reference]	18.2	1 [Reference]	17.9	1 [Reference]
30–44	25.9	0.91 (0.66–1.26)	21.1	0.99 (0.70–1.40)	18.8	0.84 (0.58–1.21)
45–59	24.9	0.84 (0.60–1.16)	13.8	0.58 (0.40–0.84)	16.4	0.68 (0.47–0.97)
≥60	22.1	0.72 (0.52–0.99)	10.4	1.12 (0.77–1.62)	10.1	0.38 (0.26–0.56)
**Race and ethnicity**						
Black or African American, non-Hispanic	18.2	0.80 (0.56–1.14)	15.9	1.12 (0.77–1.62)	15.3	1.13 (0.77–1.67)
Hispanic	23.7	1.09 (0.82–1.45)	15.1	0.96 (0.67–1.37)	16.0	1.09 (0.77–1.54)
White, non-Hispanic	24.5	1 [Reference]	15.1	1 [Reference]	14.9	1 [Reference]
≥2 races, non-Hispanic	36.7	1.88 (1.19–2.96)	21.2	1.42 (0.79–2.55)	22.5	1.60 (0.94–2.73)
Other race, non-Hispanic	24.8	0.91 (0.62–1.34)	18.1	1.10 (0.70–1.71)	17.5	1.10 (0.70–1.72)
**Education**						
High school graduate or less	19.1	0.65 (0.51–0.83)	13.2	0.78 (0.58–1.05)	12.3	0.77 (0.57–1.04)
Some college	25.2	0.86 (0.70–1.06)	15.8	0.92 (0.72–1.17)	17.5	1.10 (0.86–1.41)
Bachelor’s degree or higher	28.5	1 [Reference]	18.0	1 [Reference]	17.3	1 [Reference]
**Annual household income, $**						
<25,000	15.5	0.70 (0.46–1.07)	12.3	0.89 (0.55–1.44)	11.1	0.81 (0.49–1.34)
25,000–49,999	20.2	0.84 (0.63–1.11)	14.7	1.04 (0.74–1.46)	14.5	0.98 (0.70–1.37)
50,000–99,999	25.8	1.06 (0.86–1.29)	16.5	1.09 (0.85–1.40)	15.9	0.99 (0.77–1.27)
≥100,000	26.7	1 [Reference]	16.1	1 [Reference]	16.9	1 [Reference]
**Employment**						
Working	24.6	1 [Reference]	16.6	1 [Reference]	16.6	1 [Reference]
Not working	22.9	1.07 (0.86–1.33)	13.9	1.07 (0.82–1.38)	13.7	1.06 (0.81–1.38)
**Marital status**						
Married	27.8	1 [Reference]	16.6	1 [Reference]	17.3	1 [Reference]
Not married	18.7	0.62 (0.50–0.76)	14.1	0.69 (0.54–0.89)	13.0	0.61 (0.47–0.79)
**Metro status**						
Metro area	25.9	1 [Reference]	15.1	1 [Reference]	14.7	1 [Reference]
Nonmetro area	23.6	0.83 (0.64–1.09)	15.6	0.98 (0.72–1.32)	15.5	1.00 (0.73–1.37)
**Region**						
Northeast	20.3	0.72 (0.55–0.94)	14.6	0.91 (0.65–1.26)	15.4	0.95 (0.69–1.31)
Midwest	26.5	1 [Reference]	16.0	1 [Reference]	16.2	1 [Reference]
South	22.8	0.87 (0.70–1.10)	15.3	0.96 (0.73–1.27)	14.8	0.92 (0.69–1.21)
West	25.9	0.95 (0.74–1.23)	16.0	0.97 (0.70–1.34)	15.8	0.91 (0.66–1.26)

Abbreviation: NA, not applicable.

a Percentages are estimated using SummerStyles adult survey weights. Odds ratios and 95% CIs for each characteristic were adjusted for all other variables in this table.

## Discussion

Cancer, heart disease, and diabetes are leading causes of illness and death among US women (6). Despite its numerous health benefits, breastfeeding rates in the US are low compared with Healthy People 2030 goals (7,8). Many factors impact mothers’ feeding decisions, including family support, public perceptions, and social norms (9). Better understanding public opinions around breastfeeding could help tailor public awareness messaging and contribute to increasing breastfeeding rates and reducing subsequent burden from related chronic diseases.

Sparse literature exists on public knowledge of breastfeeding’s benefits for maternal health. However, the breast cancer literature indicates significant knowledge gaps (4,10) that vary by race and ethnicity (10,11), and that reducing knowledge gaps might increase breastfeeding rates (10).

Our study found that a low proportion of respondents believed that breastfeeding protects against any of the 3 maternal outcomes explored; most were neutral toward statements about the health benefits. These findings indicate lack of awareness or potential misunderstanding of the links between breastfeeding and maternal health. Male gender, older age, and not being married were key factors associated with less belief in the maternal benefits.

The Surgeon General’s 2020 Call to Action to Improve Maternal Health emphasizes breastfeeding support at the individual and community levels as part of a comprehensive strategy to improve women’s health (12). Strategies to educate the public on breastfeeding benefits for infants can be expanded to also emphasize maternal benefits. Increased awareness could impact mothers’ decisions to breastfeed (10) and build support for breastfeeding-friendly policies and programs, such as workplace supports and public lactation spaces. The Special Supplemental Nutrition Program for Women, Infants, and Children (WIC) program also offers opportunities for increased messaging to families and the community around maternal benefits of breastfeeding.

This study’s strengths include its use of a large diverse panel weighted to the distribution of noninstitutionalized US adults. It adds to the limited literature on public awareness of maternal benefits of breastfeeding. A main limitation is that opinions of participants may differ from nonparticipants. The survey also does not assess whether respondents were mothers, so awareness of health benefits among the population affected could not be examined.

Suboptimal breastfeeding in the US contributes to excess illness and death from maternal and pediatric disease and leads to substantial financial and nonfinancial costs (13). Those working in chronic disease, maternal and child health, and nutrition could consider strengthening partnerships and increasing messaging around the maternal benefits of breastfeeding.
